# ARE THERE ADVANTAGES IN DOUBLE TRANSIT RECONSTRUCTION AFTER TOTAL GASTRECTOMY IN PATIENTS WITH GASTRIC CANCER? A SYSTEMATIC REVIEW

**DOI:** 10.1590/0102-672020240006e1799

**Published:** 2024-05-13

**Authors:** Luigi Carlo da Silva COSTA, Ary Augusto de Castro MACEDO, Juliana Mattei de ARAÚJO, Ewerton Lima da SILVA, Luís Felipe Gomes Reis de MORAES, Aline dos SANTOS, Hugo Gomes SOARES, Valdir TERCIOTI, João de Souza COELHO, Nelson Adami ANDREOLLO, Luiz Roberto LOPES

**Affiliations:** 1 Universidade Estadual de Campinas, Faculty of Medical Sciences, Postgraduate Program in Surgical Sciences – Campinas (SP), Brazil; 2 Universidade do Estado do Pará, Medical School – Marabá (PA), Brazil; 3 Universidade do Estado do Pará, Medical School, Laboratory of Surgical Skills – Marabá (PA) – Brazil; 4 Universidade Estadual de Campinas, Faculty of Medical Sciences, Department of Surgery – Campinas (SP), Brazil

**Keywords:** Gastric Cancer, Gastrectomy, Gastric Bypass, Anastomosis in Roux-en-Y, Câncer Gástrico, Gastrectomia, Bypass Gástrico, Anastomose em Y-de-Roux

## Abstract

**BACKGROUND:**

Curative treatment for gastric cancer involves tumor resection, followed by transit reconstruction, with Roux-en-Y being the main technique employed. To permit food transit to the duodenum, which is absent in Roux-en-Y, double transit reconstruction has been used, whose theoretical advantages seem to surpass the previous technique.

**AIMS:**

To compare the clinical evolution of gastric cancer patients who underwent total gastrectomy with Roux-en-Y and double tract reconstruction.

**METHODS:**

A systematic review was carried out on Web of Science, Scopus, EmbasE, SciELO, Virtual Health Library, PubMed, Cochrane, and Google Scholar databases. Data were collected until June 11, 2022. Observational studies or clinical trials evaluating patients submitted to double tract (DT) and Roux-en-Y (RY) reconstructions were included. There was no temporal or language restriction. Review articles, case reports, case series, and incomplete texts were excluded. The risk of bias was calculated using the Cochrane tool designed for randomized clinical trials.

**RESULTS:**

Four studies of good methodological quality were included, encompassing 209 participants. In the RY group, there was a greater reduction in food intake. In the DT group, the decrease in body mass index was less pronounced compared to preoperative values.

**CONCLUSIONS:**

The double tract reconstruction had better outcomes concerning body mass index and the time until starting a light diet; however, it did not present any advantages in relation to nutritional deficits, quality of life, and post-surgical complications.

## RESUMO

**RACIONAL:**

O tratamento curativo do câncer gástrico envolve a ressecção do tumor, seguida de reconstrução do trânsito, sendo o Y-de-Roux a principal técnica empregada. Para permitir o trânsito alimentar para o duodeno, ausente em Y-de-Roux, tem-se utilizado a reconstrução de duplo trânsito, cujas vantagens teóricas parecem superar a técnica anterior.

**OBJETIVOS:**

Comparar a evolução clínica de pacientes com câncer gástrico submetidos à gastrectomia total com Y-de-Roux e reconstrução de duplo trânsito.

**MÉTODOS:**

Foi realizada uma revisão sistemática nas bases de dados: Web of Science, Scopus, Embase, Scielo, Biblioteca Virtual em Saúde, PubMed e Cochrane. Os dados foram coletados até 11 de junho de 2022. Foram incluídos estudos observacionais ou ensaios clínicos avaliando pacientes que utilizaram reconstruções de duplo trânsito (DT) e Y-de-Roux (RY). Não houve restrição temporal ou de idioma. Foram excluídos artigos de revisão, relatos de casos, séries de casos e aqueles com texto incompleto. O risco de viés foi calculado utilizando a ferramenta Cochrane desenvolvida para ensaios clínicos randomizados.

**RESULTADOS:**

Foram incluídos quatro estudos de boa qualidade metodológica, abrangendo 209 participantes. No grupo RY houve maior redução na ingestão alimentar. No grupo DT, a diminuição do índice de massa corporal (IMC) foi menos pronunciada em comparação aos valores pré-operatórios.

**CONCLUSÕES:**

A reconstrução de duplo trânsito apresentou melhores resultados em relação ao índice de massa corporal e ao tempo para início de dieta leve, porém não apresentou vantagens em relação aos déficits nutricionais, qualidade de vida e complicações pós-cirúrgicas.

## INTRODUCTION

Despite the decline observed in the last decades, gastric cancer is still the fifth most common neoplasia in the entire world (1.089.103 new cases in 2020) and represents the fourth cause of death, with 768.793 casualties in 2020^31,36^. Most patients with initial gastric cancer present symptoms of low specificity such as epigastralgia, thus making screening essential to diagnose still with a possibility of cure by surgical or endoscopic resection^35^.

Gastric cancer surgical treatment depends on the location of the tumor. Total gastrectomy is the procedure of choice for diffuse gastric cancer, gastric cancer in the upper third of the stomach and, in some cases, in the middle third. Complications may include reflux esophagitis, fistulas, and dehiscence of the esophago-jejunal anastomosis. Additionally, a great nutritional impact is observed including weight loss, malnutrition, and hypovitaminosis, which adversely affects the long-term quality of life for this group of patients^2,12^.

**Figure 1:**
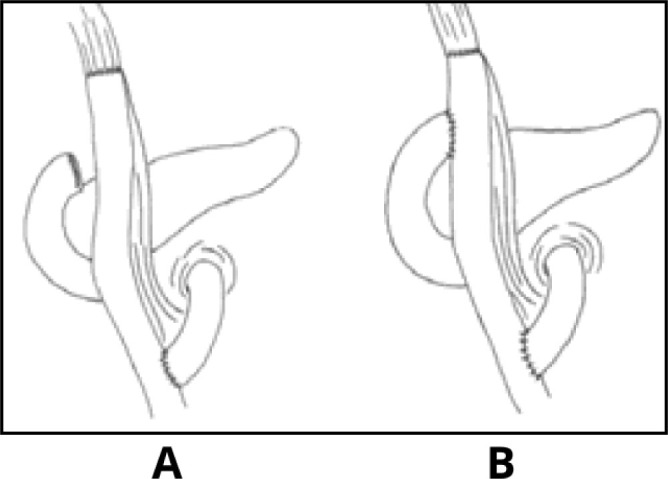
Gastric reconstruction techniques after total gastrectomy (A: Roux-en-Y; B: Double tract); adapted from Lopes et al.^18^

After resection, there are many techniques for reconstructing the digestive transit, being Roux-en-Y (RY) the most commonly used and described in the literature. This procedure involves creating an esophago-jejunal anastomosis, followed by an anastomosis of the biliopancreatic loop 40–60 cm from the esophagojejunostomy through an enteroenteroanastomosis, forming the RY configuration^17^ (Figure 1A).

**Figure 2:**
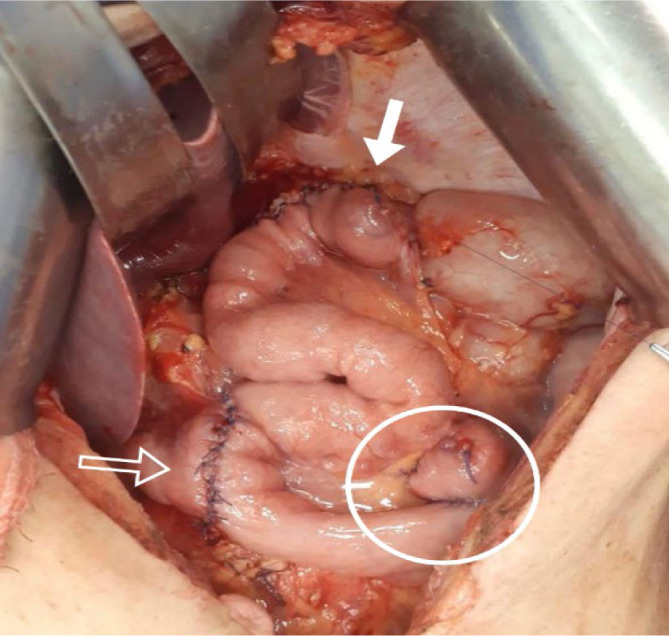
Double tract gastric reconstruction technique after total gastrectomy. (Bold white arrow: esophago-jejunal anastomosis; hollow white arrow: jejuno-duodenal anastomosis; white circle: enteroenteroanastomosis).

The alimentary transit through the duodenum can be accomplished through double transit (DT) reconstruction, starting with the RY reconstruction, followed by a side-end jejuno-duodenal anastomosis^18^ (Figure 1B and Figure 2). This technique has proven to be effective in reducing reflux symptoms and mixing bile and pancreatic juice with food, improving digestion and absorption. By allowing passage through the duodenum, it facilitates the investigation and treatment of biliary diseases that require endoscopic intervention, which is quite common in gastrectomized individuals^7,22^.

Despite the theoretical advantages of the DT technique over the RY, there is no consensus in the literature regarding the best method for gastrointestinal reconstruction after total gastrectomy in oncologic patients. Proximal gastrectomy with antral preservation in individuals with tumors in the gastric fundus and body, and reconstruction preserving alimentary transit through the duodenum have been more frequently employed by authors recently. Reviews have shown many advantages, such as safety, better postoperative recovery, better food intake, and maintenance of body weight^7,37^.

However, studies comparing the two techniques in totally gastrectomized patients, in terms of one being superior to the other, are scarce, especially regarding nutritional impact and long-term quality of life. Therefore, this systematic review aimed at comparing the clinical outcomes of gastric cancer patients who underwent total gastrectomy using RY and DT reconstructions.

## METHODS

### Search strategy

The present systematic review conforms to the recommendations and criteria outlined in the Preferred Reporting Items for Systematic Reviews and Meta-analyses (PRISMA)^20^ and the Cochrane Handbook^6^.

The guiding question was: Can reconstruction using the RY technique be replaced by the DT technique concerning the clinical outcomes of gastric cancer patients undergoing total gastrectomy?

### Data sources

The following databases were used to search for articles: Web of Science, Scopus, Embase, SciELO, Virtual Health Library (*Biblioteca Virtual em Saúde* [BVS] in Portuguese), PubMed, Cochrane, and Google Scholar. No temporal or language filters were applied, and grey literature was not considered. Data collection was conducted until June 11, 2022.

The keywords, based on the guiding question and the objectives of the problem, were selected from Health Sciences Descriptors (DeCS) by BVS and the Medical Subject Headings (MESH) terms employed by the other databases. The search terms used were: “Gastric Cancer”, “Gastrectomy”, “Double Transit Method”, and “Roux-en-Y”, each with their respective synonyms.

### Inclusion and exclusion criteria

The studies were exported to the Rayyan platform, where they underwent independent evaluation by two authors using eligibility criteria. The inclusion criteria adopted were: observational studies or clinical trials; assessment of patients who underwent the DT or RY method after total gastrectomy; and evaluation of clinical outcomes. Review articles, case reports, case series, and incomplete texts were excluded.

### Data extraction

Two researchers independently used Microsoft Excel to catalog the following data from selected studies into a spreadsheet: authors, country, sample size, age, gender, education, and outcomes. Data extraction was performed by completing a form, and thereafter, a PRISMA diagram of the selection process was created. Outcome data from the studies were then synthesized and grouped into the categories: “Clinicopathological Characteristics” when related to cancer staging; “Perioperative Procedures and Outcomes” when referring to the performed technique and aspects directly related to its execution; “Postoperative Outcomes” concerning the immediate postoperative period, such as length of hospital stay| and time until diet reintroduction; and “Nutritional Outcomes” when related to long-term nutritional changes, such as variation in body mass index (BMI). The selected data for presentation and comparison were those presented as variables in all included studies, even if there were accidental differences such as disparities in the evaluation period of the variables. The results of each article were organized into tables according to the categorization of the review.

### Risk of bias assessment

It was decided to assess the risk of bias using the Cochrane tool designed for randomized clinical trials, RoB2^6^. A table was created in Microsoft Excel 2019^®^ with the six domains evaluated by the scale and their respective scores. Then, the characteristics of each domain were blindly filled in and matched by Ewerton Lima da Silva, Luís Felipe Gomes Reis de Moraes, and Juliana Mattei de Araújo, with conflicts resolved by Luigi Carlo da Silva Costa and others.

## RESULTS

### Study selection and evaluation

**Figure 3:**
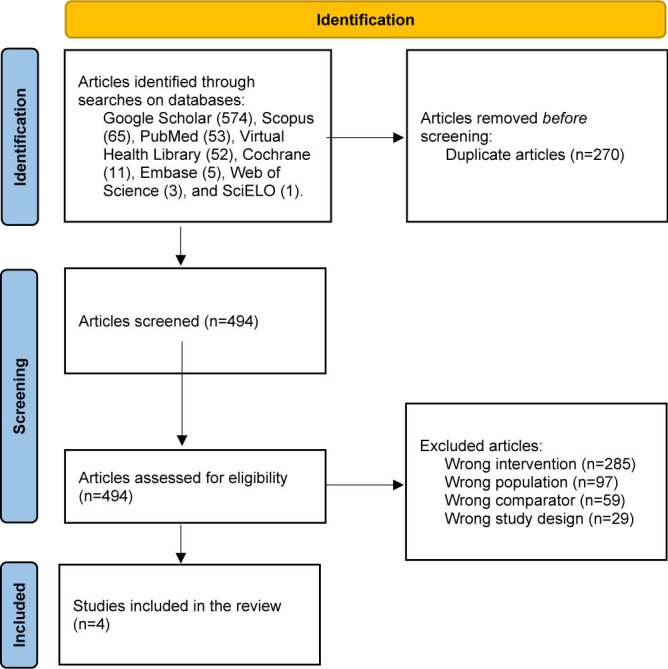
PRISMA flow diagram of the records.

The initial search identified 764 potential studies on clinical outcomes of patients undergoing the DT method compared to RY for intestinal transit reconstruction after total gastrectomy. Subsequently, duplicates were removed, resulting in 494 articles. Later, 490 studies were excluded after a detailed full-text reading for not meeting the inclusion criteria. Finally, this systematic review resulted in the inclusion of four studies (Figure 3).

PRISMA: Preferred Reporting Items for Systematic Reviews and Meta-analyses

### Included studies

The four included articles encompass a total of 209 gastric cancer patients, of whom 105 underwent reconstruction by the DT method and 104 by the RY method. Two of the studies were conducted in Serbia, one in Japan, and one in South Korea. Regarding demographic characteristics, male gender was predominantly represented in all studies, while patient age was significant only in the study by Iwahashi et al.^11^. All patients were over 18 years old.

### Methodological quality of the selected studies

The quality of the articles was analyzed through the tool Risk of Bias 2 (RoB2) by Cochrane^6^, as shown in Table 1. This tool assesses randomized studies and is organized into domains with questions aimed at obtaining important information to assess the risk of bias. The algorithm generates a judgment based on the answers to the proposed questions, with possible results being “low risk of bias”, “high risk of bias”, or “some concerns”. The studies by Iwahashi et al.^11^ and Resanovic et al.^25^ demonstrated excellent methodological quality, given the low risk of bias in the five evaluated domains. However, in the articles by Nebojša et al.^10^ and Seo et al.^28^, there were “some concerns” regarding the randomization and intervention processes.

**Table 1:** Characteristics of the included studies.

Identification			Interventions	Characteristics of the sample			Risk of Bias (RoB2)
Authors	Country	Year		n	Age (years)	Sex (M/F)	
Iwahashi et al.^11^	Japan	2009	TG+DT	21	58.2±10.7*	14/7	Low
			TG+RY	23	65.4±8.3*	18/5	
Resanovic et al.^25^	Serbia	2018	TG+DT	59	60.2±9.9	30/29	Low
			TG+RY	51	61.6±9.5	40/11	
Seo et al.^28^	Korea	2007	TG+DT	10	58.0±6.2	8/2*	Some concerns
			TG+RY	15	59.3±13.8	12/3*	
Nebojša et al.^10^	Serbia	2017	TG+DT	15	60.6±13.1	11/4	Some concerns
			TG+RY	15	65.3±6.5	10/5	

n: sample size; M: male; F: female; RoB2: Cochrane risk of bias 2; TG: total gastrectomy; DT: double transit; RY: Roux-en-Y.

* Statistical significance.

### Clinicopathological characteristics

The different classifications used to measure tumor stage were the General Rules for Gastric Cancer Study in Surgery and Pathology in Japan, the American Joint Committee on Cancer (AJCC), and the TNM classification. Statistical significance was observed by Resanovic et al.^25^, where most cases of stage IIA underwent total gastrectomy followed by reconstruction using the DT method.

Other clinicopathological characteristics such as tumor depth, lymph node metastasis, and preoperative comorbidities were not statistically significant in the studies analyzed^10,11,25,28^, as illustrated in Table 2.

**Table 2:** Clinicopathological characteristics of the included studies.

Authors	Interventions	n	Tumor Depth (T1/T2/T3)	Lymph node metastasis (-/+)	Comorbidities (+/-)
Iwahashi et al.^11^	TG+DT	21	9/8/4	16/5	DNS
	TG+RY	23	10/10/3	16/7	
Resanovic et al.^25^	TG+DT	59	NA	NA	DNS
	TG+RY	51			
Seo et al.^28^	TG+DT	10	NA	NA	NA
	TG+RY	15			
Nebojša et al.^10^	TG+DT	15	NA	NA	NA
	TG+RY	15			

n: sample size; TG: total gastrectomy; DT: double transit ; RY: Roux-en-Y; DNS: data not shown; NA: not analyzed in the study.

The Japanese study by Iwahashi et al.^11^ demonstrated a higher distribution of tumor invasion at T1 and T2 levels for both gastrointestinal tract reconstructions, with a significant absence of lymph node metastases regardless of the reconstruction technique used.

### Perioperative procedures and outcomes

The RY reconstruction was significantly faster than the DT method according to reports by Resanovic et al.^25^ (193.41±13.87 min vs. 216.01±12.89 min) and Seo et al.^28^ (248.00±16.00 min. vs. 282.00±30.00 min.). The average time for the DT reconstruction was also longer as stated by Nebojša et al.^10^ (179.60±10.15 min. vs. 178.13±11.87 min.), but on the contrary, in Iwahashi et al.’s study^11^, it was shorter (254.00±43.00 min vs. 260.00±69.00 min).

No statistical significance was observed regarding lymph node dissection technique^11^, blood loss during surgery^11^, and splenectomy^11,28^ in the analyzed studies (Table 3).

**Table 3:** Perioperative procedures and outcomes of the included studies.

Authors	Interventions	n	Surgery length (min)	Blood loss (mL)	Lymph node dissection (D1/D2)	Splenectomy (+/-)
Iwahashi et al.^11^	TG+DT	21	254.0±43.0	538±456	8/13	12/9
	TG+RY	23	260.0±69.0	513±447	5/18	14/9
Resanovic et al.^25^	TG+DT	59	216.0±12.9*	NA	0/59	NA
	TG+RY	51	193.4±13.9*		0/51	
Seo et al.^28^	TG+DT	10	282.0±30.0*	NA	0/10	3/7
	TG+RY	15	248.0±16.0*		0/15	4/11
Nebojša et al.^10^	TG+DT	15	178.1±11.9	NA	NA	NA
	TG+RY	15	179.6±10.2			

n: sample size; TG: total gastrectomy; DT: double transit; RY: Roux-en-Y; NA: not analyzed in the study.

* Statistical significance.

In the study by Iwahashi et al.^11^, more splenectomies were performed in association with the total gastrectomy compared to the results of Seo et al.^28^. Regarding lymph node dissection, Resanovic et al.^25^ and Seo et al.^28^ employed only D2 dissection, while Iwahashi et al.^11^ prioritized D1 dissection*.*

### Postoperative outcomes

Analyzing the postoperative hospital stay duration, there was no statistical significance among the authors (Table 4). The incidence of complications was assessed by Resanovic et al.^25^ and Seo et al.^28^, and the results were not statistically significant either.

**Table 4:** Postoperative nutritional outcomes of the included studies.

Authors	Interventions	Time until light diet (days)	Postoperative hospital stay (days)	Food intake decline (%)		
				3 months	1 year	3 years
Iwahashi et al.^11^	TG+DT	NA	20.70±9.90	67.50	DNS	NA
	TG+RY		20.90±8.10	64.50		
Resanovic et al.^25^	TG+DT	5.73±2.13*	NA	65.94	DNS	NA
	TG+RY	6.82±2.33*		61.64		
Seo et al.^28^	TG+DT	NA	NA	NA	74±11	91±8.8
	TG+RY				72±13	83±20
Nebojša et al.^10^	TG+DT	NA	13.20±1.26	NA	NA	NA
	TG+RY		13.07±0.88			

TG: total gastrectomy; DT: double transit ; RY: Roux-en-Y; NA: not analyzed in the study; DNS: data not shown.

* Statistical significance.

### Nutritional outcomes

The time until the introduction of a light diet, studied only by Resanovic et al.^25^, was significantly longer for the RY group (6.82±2.33 days *vs*. 5.73±2.13 days). The other studies did not analyze the number of days until the reintroduction of a light oral diet after surgery (Table 4).

Food intake decreased during the postoperative follow-up period. At three-month follow-up, patients who underwent RY reconstruction showed a reduction in food intake according to Iwahashi et al.^11^ and Resanovic et al.^25^, with decreases to 67.5% and 65.9% in the DT group and 64.5% and 61.6% in the RY group, respectively. The same parameter was evaluated by Seo et al.^28^, but within a one-year follow-up, detecting a decrease to 74.0% for the DT group and 72.0% for the RY group.

All studies assessed postoperative BMI (Table 5). After a one-year follow-up, the average BMI was statistically higher in the DT group (22.55±1.58 kg/m² vs. 21.14±1.64 kg/m²) as related by Resanovic et al.^25^, Iwahashi et al.^11^, and Seo et al.^28^, meaning that the BMI of the RY group had a greater reduction when compared to the preoperative BMI. Nebojša et al.^10^ reported no statistical significance between the preoperative and postoperative BMI; however, the RY reconstruction group had a reduction, while the DT group had an increase.

**Table 5:** Body mass index postoperative outcomes of the included studies.

Authors	Interventions	Preoperative BMI (kg/m^2^)	Postoperative BMI (kg/m^2^)			
			3 months	6 months	1 year	2 years
Iwahashi et al.^11^	TG+DT	DNS	DNS	DNS	77.8%	NA
	TG+RY				70.0%	
Resanovic et al.^25^	TG+DT	25.39±1.36	DNS	DNS	22.55±1.58*	NA
	TG+RY	25.24±1.65			21.14±1.64*	
Seo et al.^28^	TG+DT	DNS	NA	NA	89.7%	91.8%
	TG+RY				89.6%	91%
Nebojša et al.^10^	TG+DT	22.9±1.2	22.6±1.1	22.9±1.1	23.6±1.1	NA
	TG+RY	23.0±2.1	22.2±1.7	22.3±1.6	22.5±1.6	

BMI: body mass index; TG: Total gastrectomy; DT: Double transit; RY: Roux-en-Y; NA: not analyzed in the study; DNS: data not shown.

* Statistical significance.

After the surgical procedures, the quality of life was examined by different methods by Iwahashi et al.^11^ and Resanovic et al.^25^, and both identified no statistical significance. Iwahashi et al.^11^ assessed the quality of life at two time points, with lower quality of life in the RY group after three months of follow-up (37.80±6.30 vs. 36.60±5.30), which reversed after one year, showing a better outcome for this group (41.00±5.60 vs. 38.20±4.90).

The significant serum nutritional parameters evaluated by Seo et al.^28^ were total serum protein and albumin, with lower values found in the group that underwent DT reconstruction. Iwahashi et al.^11^ considered in their studies the analysis of retinol-binding serum proteins, triglycerides, calcium, iron, and immunoglobulin fractions at three months, six months, and one year postoperatively, recording similar values between the groups. The remaining studies did not assess these parameters.

## DISCUSSION

The articles included in this review are recent publications in the consulted literature, involving randomized or comparative studies on reconstruction techniques after total gastrectomy. These studies predominantly involved male patients, with an average age of 61 years, which, in agreement with the literature, is a population with a higher prevalence of gastric cancer^5,30^. Most patients were already diagnosed at an advanced stage, with approximately one-fifth presenting with metastases at their initial presentation^30^.

In Japan, a distinct pattern of gastric cancer patients is observed. In the disease diagnosis, 86.3% of patients presented tumors in early stages (I and II), probably due to the extensive screening for this cancer^11^. Early diagnosis enables the implementation of curative treatment, either surgical or endoscopic, in a larger population segment, leading to an increase in the survival rate^23^.

The traditional curative treatment involves gastrectomy associated with lymphadenectomy due to the high incidence of lymph node involvement^3,8,24^. The dissection of lymph nodes at the D1/D1+ level, by removing only perigastric lymph nodes, is a less radical strategy used in the Japanese cases in this review. Lymphadenectomy at the D2 level was the most commonly employed and involves a more extensive resection of lymph node chains along the celiac trunk, hepatic hilum, and splenic hilum, and spleen removal can also be performed^19,24^.

Over the years, more than 50 reconstruction models of the digestive tract after total stomach excision were proposed by various authors, indicating, on the one hand, the rich imagination of surgeons, but on the other hand, that there is no technique with universal acceptance. Cesar Roux deserves credit as in 1893 he described the RY reconstruction which is currently the most widely used by surgeons on all continents^2,17,18^.

It is still a controversial issue in clinical research, though, whether reconstruction after total gastrectomy for gastric cancer should be done with or without some type of reservoir or placement of the duodenum in the alimentary transit^7,17,18,37^.

The oldest reference found in the literature regarding the placement of the duodenum in the alimentary transit after total gastrectomy dates from 1958. It was a technique described by Rosanov, a Russian surgeon, performing reconstruction in RY with a latero-terminal jejuno-duodenal anastomosis^18^. Subsequently, in 1965, Kajitani et al.^13^ demonstrated the advantages of the technique in Japan. Moricca^21^ described a similar procedure in Italy, in 1976, emphasizing its simplicity and complication-free nature. In Brazil, Safatle^26^ proposed the isoperistaltic duodeno-jejunal pouch technique in 1984. However, few authors have used the technique and described their results^9,27,34^.

Lopes et al.^18^, in 2011, conducted laboratory and clinical evaluations on 43 patients who underwent total gastrectomy six months postoperatively. Among them, 32 had RY reconstruction, 11 underwent a modified Rosanov technique, a type of DT reconstruction, and 22 individuals served as a control group without surgery. Measurements included hematocrit and hemoglobin levels, serum iron, ferritin, and steatocrit of serum albumin. No postoperative complications were recorded in this casuistry. Clinical assessments investigated BMI, nausea and vomiting, heartburn, reflux, postprandial abdominal distension, anorexia, and daily number of evacuations. The authors concluded that preserving duodenal transit offers advantages such as better mixing of food with enzymes, increased fat absorption, lower prevalence of symptoms like abdominal distension, diarrhea, heartburn, and anorexia, and an improved pattern of laboratory tests.

Thus, the methods of reconstructing the digestive tract after total gastrectomy aim to maintain a natural and adequate food passage, providing postoperative quality of life for patients in the short, medium, and long terms^37^.

The evaluated reconstruction techniques, DT and RY, have not yet addressed the nutritional deficiency after total gastrectomy. Gastric cancer itself leads to malnutrition due to anorexia, which can occur due to mechanical obstruction by the tumor or even a state of cachexia, which involves the exaggerated release of pro-inflammatory cytokines and leptin dysregulation^33^. Weight loss is more pronounced after total gastrectomy because the stomach’s physiological functions of storage and digestion are impaired, reducing food intake and consequently BMI^15,16^.

The studies herein agree that the decrease in BMI is a constant after total gastrectomy, with greater declines observed in RY reconstruction compared to DT. However, the Korean study observed that patients who underwent RY reconstruction exhibited higher levels of albumin and total serum proteins three years after the procedure^28^. Regarding the time until the reintroduction of a light diet, an outcome assessed by Resanovic et al.^25^, DT reconstruction proved to be superior, having a shorter period.

DT reconstruction also involves ease of subsequent access to the duodenum and the biliopancreatic system, facilitating the treatment of biliary complications that may be related to disease progression^1,22^. There are techniques for duodenal access after RY reconstruction, such as single and double balloon enteroscopy. However, the altered anatomy and the reversed angle of view compared to the usual make it challenging to perform procedures in the biliopancreatic system when compared to endoscopic retrograde cholangiopancreatography^32^.

The reconstruction of the biliopancreatic loop presents benefits regarding appetite and in preventing the development of postprandial hyperglycemia, commonly observed in the RY anastomosis due to the rapid increase in hormones such as insulin, cholecystokinin, and somatostatin after meals^14^. Kalmár et al.^14^, studying glucose metabolism and these hormones in total gastrectomized patients, found that glucose metabolism disorders are more evident in RY reconstruction. Additionally, the response of cholecystokinin and somatostatin differed significantly in favor of preserving duodenal alimentary transit after total gastrectomy. It was concluded that levels of cholecystokinin close to physiological ones found in alimentary reconstruction with the duodenum may contribute to preserved physiological satiety after total gastrectomy^14^.

By promoting better BMI and body weight levels, the DT reconstruction assists in maintaining body weight, as demonstrated by the evidence^3^. Such observation can be justified by preserving a physiological duodenal pathway, which favors the hormonal regulation and the mixing of bile and pancreatic juices with ingested food, optimizing absorption and microbiota control^37^.

Malabsorption by the digestive tract can be assessed through steatorrhea, a common finding in patients undergoing total gastrectomy^4^. The evaluation of fecal steatocrit is an easy way to estimate digestion and absorption in the tract, as suggested by an experimental study conducted on rats subjected to total gastrectomy followed by tract reconstruction. The control group had fecal steatocrit values similar to those with double transit, 4.14% and 4.46% respectively, while the mean for the RY reconstruction group was 28.17%^29^.

Regarding the aspects related to the performed procedure, differences and disagreements were found among the included studies. Those conducted in Serbia agreed that the RY reconstruction technique was significantly faster than the DT^10,25^. However, the work of Iwahashi et al.^11^ in Japan yielded results showing a longer reconstruction time for RY, although the difference was not statistically significant.

This review has limitations that must be taken into account when extrapolating conclusions to clinical practice, as it considered only four studies. Additionally, the populations evaluated in the studies were predominantly Serbian patients, with the remaining divided between Koreans and Japanese, making them distinct in terms of demographic, cultural, and genetic factors. Furthermore, the included studies have methodological divergences that complicate the comparison of results, such as those related to quality of life, which were not assessed using the same tools, the investigation of different biochemical outcomes, and the variation in the periods selected for evaluating the patients. It is important to note that concerns raised in the risk of bias assessment in some studies do not diminish the credibility of the biochemical, metabolic, and nutritional outcomes obtained.

More refined studies are needed, exploring other nutritional parameters and conducting late assessments of steatorrhea, such as fecal steatocrit and other measures of the absorptive capacity of the gastrointestinal tract. This is essential for the formation of a more robust body of evidence capable of guiding the choosing of one technique or the other, benefiting nutritional impact and quality of life.

## CONCLUSION

Although this review did not demonstrate clear results that lead us to conclude an overall improvement in nutritional aspects, the DT reconstruction after total gastrectomy showed better outcomes compared to the RY reconstruction in terms of BMI gain and an earlier start of a light diet. The DT reconstruction is a straightforward procedure that does not excessively prolong the operation time, reduces the risk of duodenal stump fistula, facilitates postoperative access to the biliary and pancreatic pathways, and allows the passage of food to the duodenum. However, the findings are not sufficient to support an effective advantage of DT, as essential postoperative aspects such as nutritional deficit, quality of life, and complications were not influenced by the type of reconstruction.
